# Multiple mechanisms mediating carbon monoxide inhibition of the voltage-gated K^+^ channel Kv1.5

**DOI:** 10.1038/cddis.2017.568

**Published:** 2017-11-02

**Authors:** Moza M Al-Owais, Nishani T Hettiarachchi, John P Boyle, Jason L Scragg, Jacobo Elies, Mark L Dallas, Jon D Lippiat, Derek S Steele, Chris Peers

**Affiliations:** 1 Division of Cardiovascular and Diabetes Research, LICAMM, Faculty of Medicine and Health, Faculty of Biological Sciences, University of Leeds, Leeds LS2 9JT, UK; 2 School of Biomedical Sciences, Faculty of Biological Sciences, University of Leeds, Leeds LS2 9JT, UK

## Abstract

The voltage-gated K^+^ channel has key roles in the vasculature and in atrial excitability and contributes to apoptosis in various tissues. In this study, we have explored its regulation by carbon monoxide (CO), a product of the cytoprotective heme oxygenase enzymes, and a recognized toxin. CO inhibited recombinant Kv1.5 expressed in HEK293 cells in a concentration-dependent manner that involved multiple signalling pathways. CO inhibition was partially reversed by superoxide dismutase mimetics and by suppression of mitochondrial reactive oxygen species. CO also elevated intracellular nitric oxide (NO) levels. Prevention of NO formation also partially reversed CO inhibition of Kv1.5, as did inhibition of soluble guanylyl cyclase. CO also elevated intracellular peroxynitrite levels, and a peroxynitrite scavenger markedly attenuated the ability of CO to inhibit Kv1.5. CO caused nitrosylation of Kv1.5, an effect that was also observed in C331A and C346A mutant forms of the channel, which had previously been suggested as nitrosylation sites within Kv1.5. Augmentation of Kv1.5 via exposure to hydrogen peroxide was fully reversed by CO. Native Kv1.5 recorded in HL-1 murine atrial cells was also inhibited by CO. Action potentials recorded in HL-1 cells were increased in amplitude and duration by CO, an effect mimicked and occluded by pharmacological inhibition of Kv1.5. Our data indicate that Kv1.5 is a target for modulation by CO via multiple mechanisms. This regulation has important implications for diverse cellular functions, including excitability, contractility and apoptosis.

Kv1.5 is a rapidly activating, voltage-gated K^+^ channel encoded by KCNA5 that inactivates slowly and incompletely.^1^ Distribution of Kv1.5 is widespread: it is expressed in various cell types in the central nervous system^2, 3^ and is implicated in certain types of cancers.^4^ Kv1.5 is, however, perhaps best studied in the cardiovascular system. Its expression/activity is associated with increased apoptosis in endothelial and smooth muscle cells.^5, 6^ In vascular smooth muscle cells (VSMCs) of the pulmonary vasculature, it is of particular importance to hypoxic pulmonary vasoconstriction^7, 8, 9^ and in the development of pulmonary arterial hypertension (PAH).^10, 11, 12^ Indeed, Kv1.5 expression is reduced in PAH patients^13^ and patients with idiopathic PAH possess important single-nucleotide polymorphisms in *KCNA5*, which encodes Kv1.5.^14, 15^ In the systemic circulation, Kv1.5 also contributes to repolarization of the VSMC membrane potential, limiting Ca^2+^ entry and hence vascular tone.^16, 17, 18^ A recent study employing Kv1.5^−/−^ mice has shown that this channel is essential for balancing coronary blood flow with metabolic demands of the working myocardium.^19^

In the heart, Kv1.5 expression is largely confined to the atria where it is responsible for the ultrarapid outward current, IK_ur_, the major repolarizing current that is active throughout phases 1–3 of the atrial action potential (AP).^20, 21^ Targeting of Kv1.5 activity/expression is currently regarded as a promising therapeutic approach to the treatment of atrial fibrillation (AF).^22, 23, 24, 25^ Given the widespread importance of Kv1.5 in the cardiovascular system and elsewhere, it is perhaps unsurprising that it is regulated via numerous posttranslational modifications, including ubiquitination,^26^ sumoylation,^26^ palmitoylation,^27^ phosphorylation^28, 29^ and nitrosylation.^30^ An additional means of regulation is via its sensitivity to reactive oxygen species (ROS). For example, tonic ROS production by mitochondria or NADPH oxidase (Nox 4) sustains Kv1.5 activity and keeps pulmonary VSMCs relatively hyperpolarized.^31, 32^ In the coronary circulation, hydrogen peroxide (H_2_O_2_) has been proposed as the signal closely coupling cardiac metabolism to coronary blood flow^33, 34, 35^ and this coupling appears via H_2_O_2_-mediated augmentation of Kv1.5.^19^ Recombinant Kv1.5 activity has also been demonstrated to be directly augmented by H_2_O_2_.^36^

An additional modulator of Kv1.5 is nitric oxide (NO), a long-established, biologically active signalling molecule in the cardiovascular system as well as other tissues.^37, 38, 39^ NO regulates Kv1.5 via nitrosylation and activation of cGMP,^30^ an effect which is of potential importance in the context of AF, given the important role of this channel in atrial electrical activity, and also the fact that NO bioavailability is reduced in AF and NO synthases (NOSs) can become uncoupled, leading to superoxide formation.^38, 40, 41^

Accumulating data continue to establish carbon monoxide (CO) as an important gasotransmitter alongside NO (and hydrogen sulphide), which acts to provide a range of beneficial cardiovascular (and other) effects. All three of these gasotransmitters are products of distinct, widely distributed enzymes.^42, 43^ CO dilates coronary and other vessels^44, 45, 46^ and induction of heme oxygenase-1 (HO-1, which produces CO) protects against, for example, myocardial infarction, hypertension and vascular injury.^47, 48^ CO accounts for many of the effects of HO-1 induction^49, 50, 51^ and CO inhalation, as well as CO-releasing molecules (CORMs), are being developed for cardiovascular therapy.^52, 53^ Importantly, HO-1 expression is increased in AF and appears to provide protection against the oxidative stress of this condition.^54, 55, 56^ Given the important role for Kv1.5 in normal atrial function, its redox sensitivity and the likely involvement of HO-1 as a means of providing protection in AF, we have explored the potential for CO-mediated regulation of Kv1.5 channels using both a recombinant expression system and murine atrial (HL-1) cells.

## Results

### CO inhibits recombinant human Kv1.5

**Figure 1:**
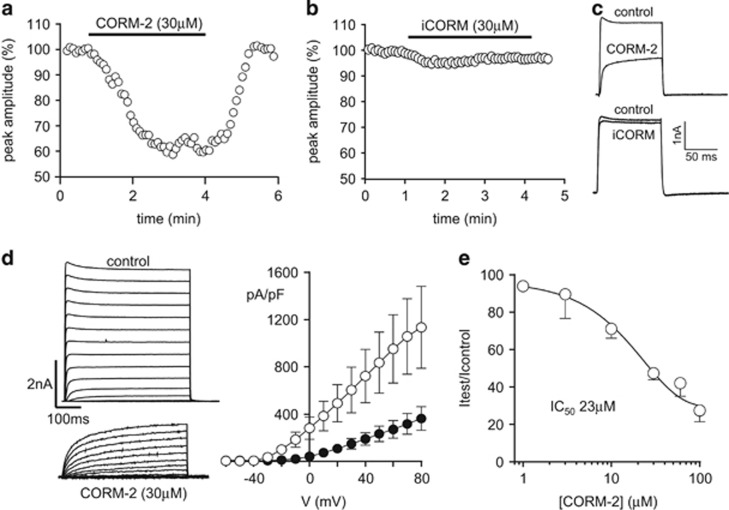
CO inhibits recombinant hKv1.5. (**a**) Time-series plot (generated by repeated step depolarizations from −80 to +50 mV (100 ms duration, 0.2 Hz)) obtained from a HEK293 cell stably expressing human Kv1.5 (hKv1.5). Plot shows normalized peak current amplitudes. For the period indicated by the horizontal bar, the cell was exposed to 30 *μ*M CORM-2. (**b**) As in panel (**a**), except this cell was exposed to 30 *μ*M iCORM (inactive breakdown product of CORM-2), as indicated. (**c**) Example currents from the plotted time series evoked before and during application of CORM-2 (upper) and iCORM (lower). (**d**) Left, currents evoked in a HEK293 cell expressing hKv1.5 before and during exposure to 30 *μ*M CORM-2, as indicated. Currents were evoked by step-depolarizations from −60 to +80 mV. Right, Mean (±S.E.M., *n*=6) current–density *versus* voltage relationships obtained before (open symbols) and during (solid symbols) application of 30 *μ*M CORM-2. (**e**) Concentration–response curve for CORM-2 inhibition of hKv1.5. Each point is the mean (±S.E.M., *n*=3–17 cells in each case) fractional inhibition caused by CORM-2. Data fit yields IC_50_ of 23 *μ*M

To examine any potential modulation of Kv1.5 by CO, we applied the CORM, CORM-2, to HEK293 cells stably expressing human Kv1.5 (hKv1.5). As exemplified in the time-series plot of Figure 1a, CORM-2 caused a reversible inhibition of K^+^ current amplitudes, and this was associated with a marked slowing of activation kinetics (Figure 1c). By contrast, the inactive form of CORM-2, iCORM, was without significant effect (Figures 1b and c). Inhibition of hKv1.5, and the associated slowing of kinetics, was seen throughout the range of activation test potentials employed (up to +80 mV; Figure 1d). A concentration–response relationship was constructed using time-series recordings as exemplified in Figure 1a, which yielded an IC_50_ value of 23 *μ*M for CORM-2 inhibition of hKv1.5 (Figure 1e).

**Figure 2:**
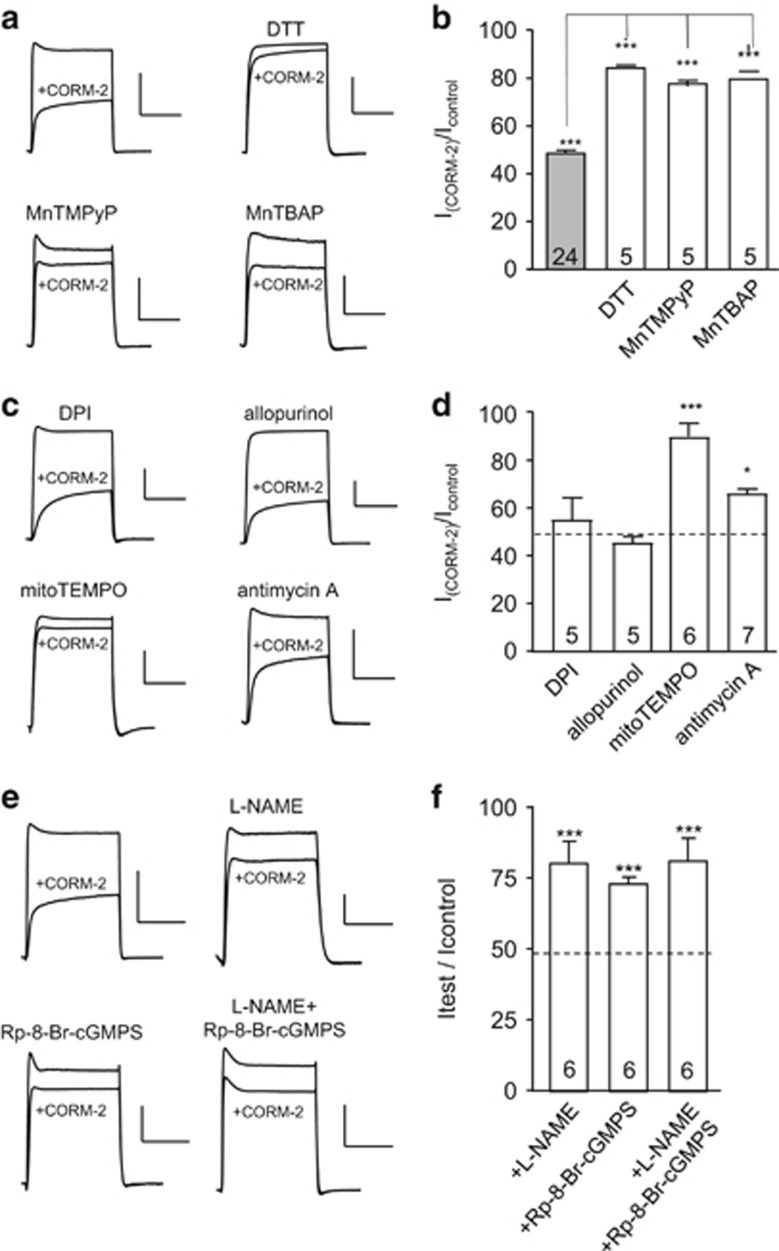
CO inhibition of hKv1.5 involves mitochondrial reactive oxygen species, NO and soluble guanylyl cyclase. (**a**) Example currents evoked in hKv1.5-expressing HEK293 cells before and during exposure to 30 *μ*M CORM-2. Currents are examples from time-series studies (as in Figure 1) in which cells were repeatedly depolarized from −80 mV to +50 mV (100 ms duration, 0.2 Hz)). CORM-2 was applied either to untreated cells or to cells pretreated for 1 h at 37 °C with DTT (1 mM) MnTMPyP (50 *μ*M) or MnTBAP (10 *μ*M), as indicated. Scale bars represent 1 nA (vertical) and 50 ms (horizontal) in all cases. (**b**) Bar graph plotting mean (with S.E.M. bars, from number of cells indicated) % inhibition caused by CORM-2 in the absence or presence of the drugs indicated (taken from studies exemplified by current traces). ***P<0.001 (effects of CORM-2 alone when compared with controls); ***P<0.001 (comparing effects of CORM-2 alone with CORM-2 in the presence of drugs indicated). (**c**) As in panel (**a**), except cells were pretreated (1 h at 37 °C) with DPI (3 *μ*M), allopurinol (1 *μ*M), mitoTEMPO (10 *μ*M) or antimycin A (3 *μ*M), as indicated, prior to recordings. (**d**) Bar graph plotting mean (with S.E.M. bars) % inhibition caused by CORM-2 in the absence or presence of the drugs indicated. **P<0.01, *P<0.05, comparing the effects of CORM-2 alone or with CORM-2 in the presence of drugs indicated. Dashed line indicates the mean effect of CORM-2 alone. (**e**) Example currents evoked in hKv1.5-expressing HEK293 cells before and during exposure to 30 *μ*M CORM-2. Currents are examples from time-series studies (as in Figure 1) in which cells were repeatedly depolarized from −80 to +50 mV (100 ms duration, 0.2 Hz)). CORM-2 was applied either to untreated cells or to cells pretreated with L-NAME (1 mM; 1 h, 37 °C), Rp-8-Br-cGMPS (50 *μ*M; 1 h, 37 °C) or both agents together, as indicated. Scale bars represent 1 nA (vertical) and 50 ms (horizontal) in all cases; except the L-NAME-treated example (vertical scale bar=0.2 nA). (**f**) Bar graph plotting mean (with S.E.M. bars, *n* numbers indicated in each bar) % inhibition caused by CORM-2 in the presence of the drugs indicated (taken from studies exemplified by current traces). Mean effect of CORM-2 alone is indicated by the dashed line. ***P<0.001, significant difference from inhibition caused by CORM-2 alone

CO can regulate ion channels via modulation of numerous signalling pathways.^57^ To investigate the mechanism of regulation of hKv1.5 by CO, we first explored the involvement of ROS. Figures 2a and b indicate that the ability of CORM-2 to inhibit hKv1.5 was significantly suppressed by a pretreatment of cells (1 h at 37 °C) with either the reducing agent dithiothreitol (DTT, 1 mM) or each of two superoxide dismutase (SOD) mimetics, 5,10,15,20-tetrakis(1-methylpyridinium-4-yl)-21*H*,23*H* porphyrin manganese (III) pentachloride (MnTMPyP; 50 *μ*M), and manganese (III) tetrakis (4-benzoic acid) porphyrin chloride (MnTBAP; 10 *μ*M). All three of these agents significantly, but incompletely, reversed the inhibitory actions of CO, strongly suggesting that ROS contributed to CO-mediated inhibition of hKv1.5. To explore the source of ROS involved in CO-mediated inhibition of Kv1.5, cells were pretreated (1 h at 37 °C) with either diphenylene iodonium (DPI; 3 *μ*M), a non-selective inhibitor of NADPH oxidases, or allopurinol (1 *μ*M), a xanthine oxidase inhibitor. Neither agent altered the ability of CORM-2 to inhibit hKv1.5 (Figures 2c and d). By contrast, pretreatment of cells with mitoTEMPO (10 *μ*M), a mitochondrially targetted antioxidant, almost fully reversed the inhibitory effects of CO (Figures 2c and d). Inhibition of hKv1.5 by CO was also significantly reduced following pretreatment with antimycin A, which inhibits complex III of the electron transport chain (Figures 2c and d). Thus mitochondria appear to be the source of ROS involved in CO-mediated inhibition of hKv1.5.

**Figure 3:**
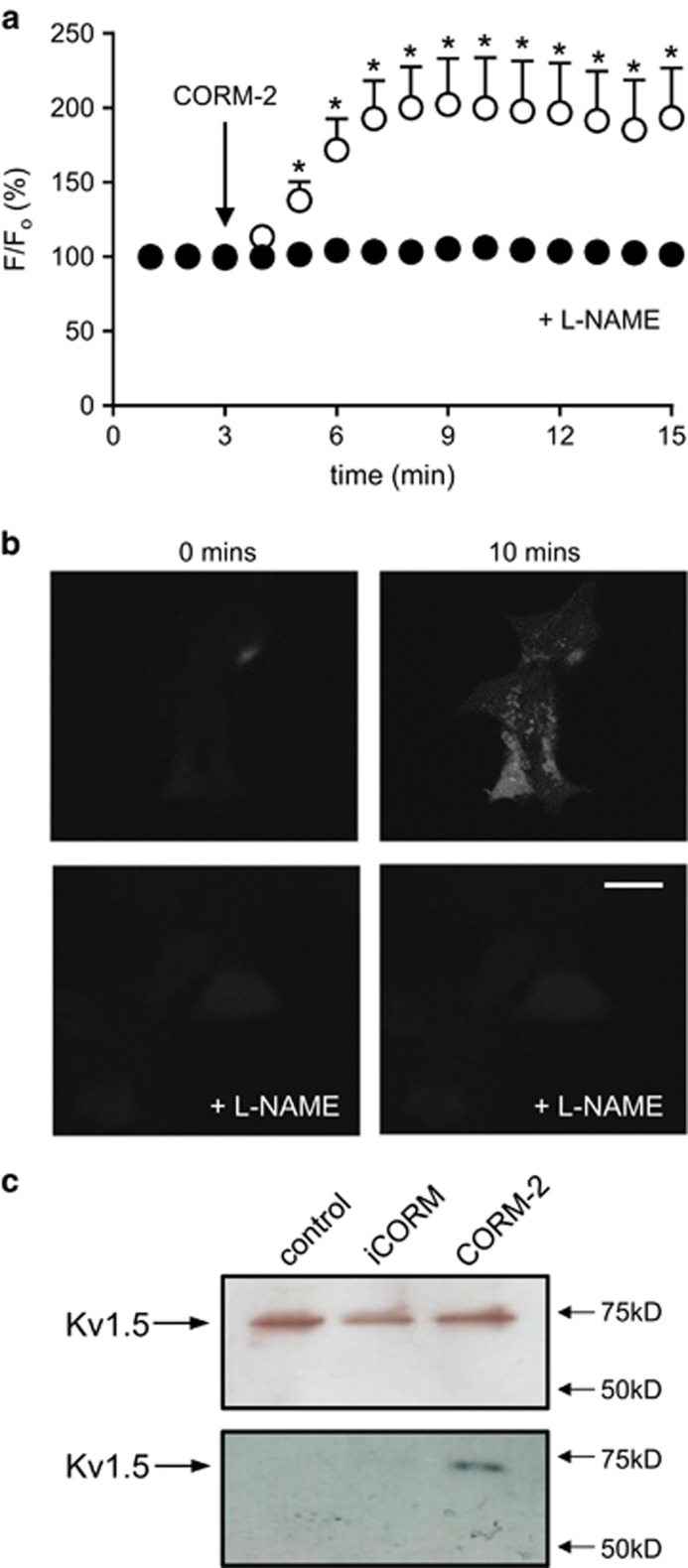
CO stimulates NO formation and nitrosylation of hKv1.5. (**a**) Mean (±S.E.M.) fluorescence recorded in cells loaded with the NO indicator, DAF-2. Fluorescence was measured in untreated (control) cells (open circles, *n*=5) or cells pretreated with L-NAME (1 mM; 1 h, 37 °C; *n*=5). (**b**) Example images at 0 and 10 min after commencement of CORM-2 application, as indicated, in untreated and L-NAME-treated cells. Scale bar (20 *μ*m) applies to all panels. (**c**) Upper; western blotting showing similar levels of hKv1.5 in cell lystates treated with CORM-2 or iCORM (30 *μ*M, 1 h). Lower, detection of nitrosylation via the biotin-switch assay. Note: nitrosylation of hKv1.5 was only detected in samples treated with CORM-2

CO is also known to activate NOS and soluble guanylyl cyclase (sGC) in several cell types. Pretreatment of cells (1 h at 37 °C) with the NOS inhibitor L-NAME (1 mM) significantly attenuated the ability of CORM-2 to inhibit hKv1.5 (Figures 2e and f). Similarly, pretreatment of cells (1 h at 37 °C) with the membrane-permeable sGC inhibitor, Rp-8-Br-cGMPS (100 nM), significantly reduced the inhibitory effects of CORM-2 on hKv1.5 (Figures 2e and f). Pretreatment of cells with both agents similarly reduced the ability of CO to inhibit currents (Figures 2e and f). These data suggested that CO could stimulate NO formation and this was further confirmed by monitoring NO levels in hKv1.5-expressing HEK293 cells using the NO-sensitive fluoroprobe, DAF-2 (Figures 3a and b). Application of CORM-2 (30 *μ*M) to DAF-2-loaded cells caused a significant rise in fluorescence, which was fully attenuated following preincubation of cells with 1 mM L-NAME (1 h, 37 °C). It is likely, therefore, that CO inhibits hKv1.5 in part via activation of NO formation, as a previous study has suggested that NO can inhibit Kv1.5.^30^ NO inhibition was shown by Nunez *et al.*^30^ to be mediated partly via PKG-dependent phosphorylation, as is the case for CO (Figure 3), and also by nitrosylation. To explore nitrosylation as a mechanism for CO-mediated inhibition of hKv1.5, we employed the biotin-switch technique and detected nitrosylation of hKv1.5 protein by CORM-2 but not by iCORM (Figure 3c), indicating that CO does indeed stimulate nitrosylation of hKv1.5.

**Figure 4:**
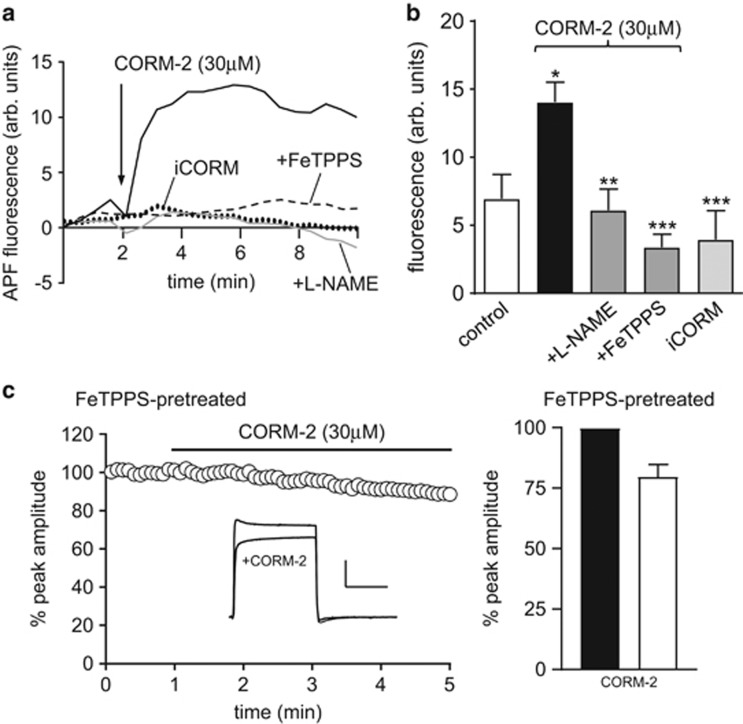
CO stimulates ONOO formation, which contributes to the inhibition of hKv1.5. (**a**) Example plots of the fluorescence levels in APF-loaded cells. At the point indicated by the arrow, cells were exposed to 30 *μ*M CORM-2 alone (solid line) or following pretreatment for 1 h at 37 °C with FeTPPS (50 *μ*M; dashed line) or L-NAME (1 mM; grey line). Also shown is the level of fluorescence detected in a cell exposed to 30 *μ*M iCORM (dotted line). (**b**) Mean level of fluorescence detected in APF-loaded cells 5 min after exposure to CORM-2 alone or in the additional presence of FeTPPS or L-NAME or when exposed to iCORM or no drugs (control). Bars represent mean±S.E.M. taken from between five and eight cells in each case. (**c**) Left, time-series plot (generated by repeated step-depolarizations from −80 to +50 mV (100 ms duration, 0.2 Hz)) obtained from a hKv1.5-expressing HEK293 cell previously exposed to 50 *μ*M FeTPPS (1 h at 37 °C). Plot shows normalized peak current amplitudes. For the period indicated by the horizontal bar, the cell was exposed to 30 *μ*M CORM-2. Inset shows example currents before and during CORM-2 exposure (scale bars: 1 nA (vertical), 50 ms (horizontal)). Right, bar graph showing the mean (±S.E.M.; *n*=5 cells) effects of CORM-2 following pretreatment with FeTPPS

The observation that CO raises ROS levels (presumably levels of superoxide, as SOD mimetics ameliorated the effects of CO; Figure 2), and also raises NO levels suggests the possibility that peroxynitrite (ONOO^−^) formation occurs in the presence of CO, as we have previously suggested.^58^ In support of this idea, we found that CO increased the level of fluorescence in cells loaded with the ONOO^−^ indicator, 2-[6-(4’-amino) phenoxy-3H-xanthen-3-on-9-yl]benzoic acid (APF; Figure 4a). These rises were fully attenuated by both L-NAME and the ONOO^−^ scavenger, FeTPPS (Figures 4a and b). Furthermore, pretreatment of cells with FeTPPS strongly attenuated the CO-mediated inhibition of hKv1.5 (Figure 4c).

### Exploring the roles of C346 and C331

**Figure 5:**
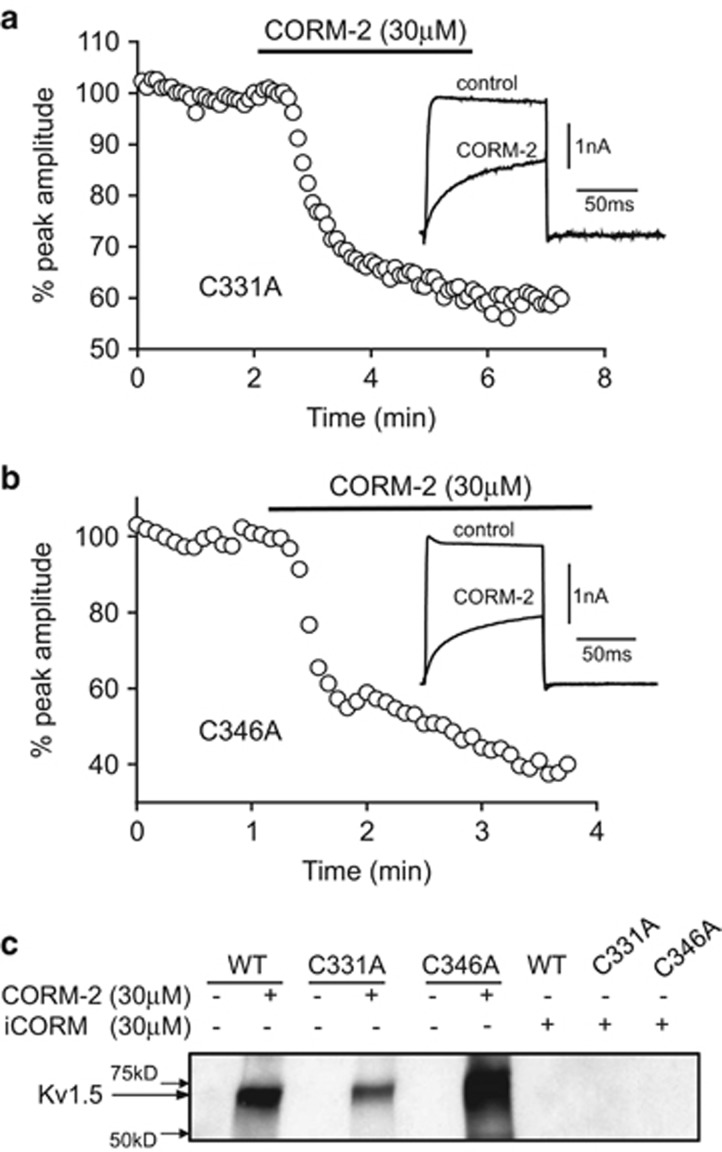
Mutation of C331 or C346 does not impede CO-mediated inhibition of hKv1.5. (**a**) Time-series plot (generated by repeated step-depolarizations from −80 to +50 mV (100 ms duration, 0.2 Hz)) obtained from a HEK293 cell stably expressing hKv1.5 containing the C331A mutation. Plot shows normalized peak current amplitudes. For the period indicated by the horizontal bar, the cell was exposed to 30 *μ*M CORM-2. Inset shows example currents recorded before and during CORM-2 application, as indicated. (**b**) As in panel (**a**), except currents were recorded from a cell stably expressing hKv1.5 containing the C346A mutation. (**c**) Nitrosylation of WT and mutant hKv1.5 channels was detected using the biotin-switch assay. Note: nitrosylation of hKv1.5 was only detected in samples treated with CORM-2, not iCORM

Based on structural modelling, it has previously been suggested (but not demonstrated) that two cysteine (C) residues within hKv1.5 might be nitrosylated by NO and thereby account for its inhibitory action on the channel.^28^ As much of the effects of CO, as reported here, are mediated by NO formation (Figures 2 and 3), we explored the potential involvement of these two residues, C331 and C346. To do this, we generated C→A (alanine) substitution mutants. As shown in Figure 5, CO was still able to inhibit the activity of the C331A (Figure 5a) and C346A mutant channels (Figure 5b). The degree of inhibition caused by 30 *μ*M CORM-2 (44±8.6%, mean±S.E.M., *n*=5, *P*<0.001 for C331A and 47±1.7%, mean±S.E.M., *n*=5, *P*<0.001 for C346A) was not significantly different from the degree of inhibition seen in the WT (non-mutant) hKv1.5 channel. Furthermore, we could detect nitrosylation of both mutants using the biotin-switch technique (Figure 5c). These findings discount both C331 and C346 as important residues in the response of hKv1.5 to CO or NO.

### CO reverses H_2_O_2_ augmentation of Kv1.5

**Figure 6:**
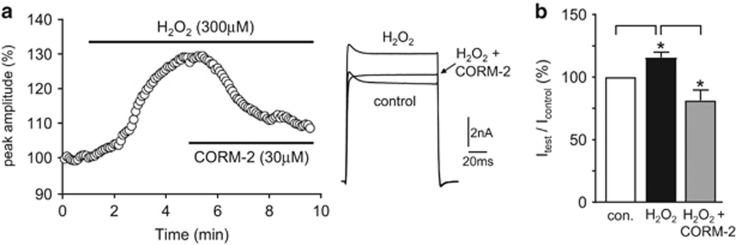
CO reverses H_2_O_2_-mediated augmentation of hKv1.5 activity. (**a**) Left, time-series plot, generated by repeated step-depolarizations from −80 to +50 mV (100 ms duration, 0.2 Hz), obtained from a HEK293 cell stably expressing hKv1.5. Plot shows normalized peak current amplitudes. For the period indicated by the upper horizontal bar, the cell was exposed to 300 *μ*M H_2_O_2_. For the period indicated by the lower bar, 30 *μ*M CORM-2 was also present. Right, example currents evoked before and during H_2_O_2_ application alone or together with CORM-2, as indicated. (**b**) Bar graph indicating the mean (±S.E.M. (*n*=5)) percentage change in current amplitude caused by H_2_O_2_ alone or together with CORM-2, as indicated. *P<0.05

The activity of both native and recombinant Kv1.5 channels has been shown to be augmented by H_2_O_2_,^32, 36^ and this has been proposed as a mechanism by which cardiac metabolism is linked to coronary blood flow.^33, 34, 35^ In agreement with these studies, we found that H_2_O_2_ (300 *μ*M) augmented Kv1.5 activity. This effect was fully reversed by CORM-2 (30 *μ*M), as exemplified in Figure 6a and quantified in Figure 6b. Given the partial prevention of the effects of CO by mitoTEMPO and SOD mimetics, this result suggests that H_2_O_2_ augmentation of Kv1.5 is an effect strikingly distinct from the formation of superoxide or ONOO^−^ that contribute to CO inhibition of the channel.

### Effects of CO in HL-1 atrial cells

**Figure 7:**
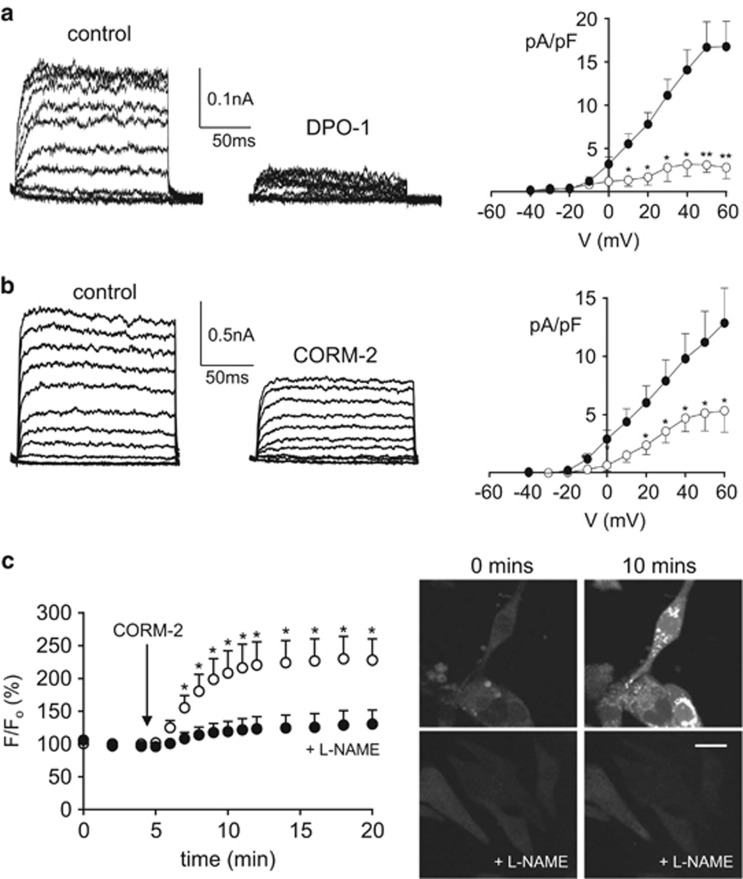
CO inhibits native K^+^ currents and raises NO levels in HL-1 atrial cells. (**a**) Families of outward K^+^ currents recorded in an example HL-1 cells by step-depolarizations as described in Methods section, before (control) and during (DPO-1) bath application of the Kv1.5 inhibitor, DPO-1 (1 *μ*M). Right, mean (±S.E.M., *n*=7 cells) current density *versus* membrane potential plot before (solid symbols) and during (open symbols) application of 1 *μ*M DPO-1. (**b**) Exactly as in panel (**a**), except cells were exposed to CORM-2 (30 *μ*M; *n*=9) rather than DPO-1. (**c**) Left, mean (±S.E.M.) fluorescence recorded in HL-1 cells loaded with the NO indicator, DAF-2. Fluorescence was measured in untreated cells (open circles, *n*=5) or cells pretreated with L-NAME (1 mM; solid circles; 1 h, 37 °C; *n*=5). Right, example images at 0 and 10 min after commencement of CORM-2 application, as indicated, in untreated and L-NAME-treated cells. Scale bar (20 *μ*m) applies to all images. In all panels, **P<0.01, *P<0.05

To examine the potential for modulation of native Kv1.5 by CO, we employed the atrial cell line, HL-1.^59^ Using a voltage protocol designed to isolate IK_ur_ (which is attributable to Kv1.5 activity), step-depolarizations evoked outward K^+^ currents that were strongly reduced in amplitude by the Kv1.5 inhibitor DPO-1 (1 *μ*M; Figure 7a).^60^ These currents were also inhibited by CORM-2 (Figure 7b), suggesting that CO could inhibit native Kv1.5 channels in HL-1 cells, as it does recombinant Kv1.5 channels. Indeed, 30 *μ*M CORM-2 caused a 49.8±5% (*n*=9) inhibition of currents at +50 mV, an effect quantitatively similar to that observed for recombinant channels (Figure 1). Furthermore, CORM-2 also evoked a measurable rise in NO levels in HL-1 cells, an effect that was effectively inhibited by L-NAME (Figure 7c), thereby suggesting an important role for NO in the actions of CO not only in HL-1 cells but also in HEK293 cells expressing hKv1.5.

**Figure 8:**
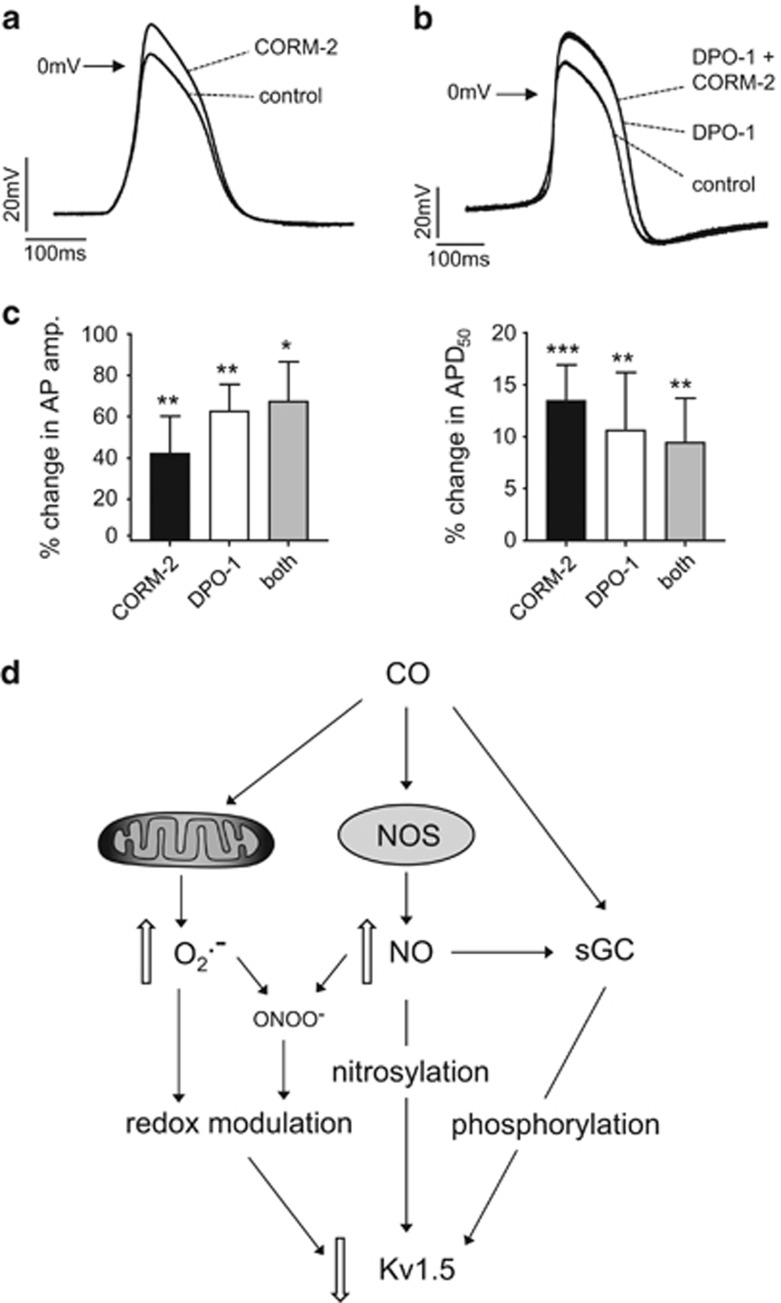
CO augments action potentials in HL-1 cells. (**a**) Spontaneous action potentials (APs) recorded in an example HL-1 cell before (control) and during application of 30 *μ*M CORM-2. Note: CORM-2 increases the AP amplitude and duration. (**b**) As in panel (**a**), except that the cell was exposed to DPO-1 (1 *μ*M), then CORM-2 (30 *μ*M) in the continued presence of DPO-1. Note: DPO-1 augments APs and prevents further augmentation by CORM-2. (**c**) Mean (±S.E.M.) augmentation in AP amplitude (AP amp.; left) and APD_50_ (right) caused by CORM-2 alone (*n*=10), DPO-1 (*n*=9) alone or CORM-2 in the continued presence of DPO-1 (both; *n*=8). Significant difference from control (no drug): ***P<0.001, **P<0.01, *P<0.05. There were no significant differences between the conditions. (**d**) Schematic mechanism for the inhibition of Kv1.5 by CO. Data presented suggest CO increases ROS (presumably superoxide, O_2_^−^) formation from mitochondria, which may directly regulate Kv1.5, but can also combine with NO (levels of which increase in response to CO) to form peroxynitrite (ONOO^−^) to cause channel inhibition. Elevated NO levels also directly nitrosylate Kv1.5. CO can also stimulate sGC (an effect that is also promoted by elevated NO levels), which leads to channel phosphorylation

Under current-clamp conditions, we also recorded spontaneous action potentials in HL-1 cells. We found that CORM-2 (30 *μ*M) significantly increased action potential amplitudes and also increased their duration (Figures 8a and c). Importantly, a similar effect was seen when HL-1 cells were exposed to DPO-1 (Figures 8b and c). Furthermore, in the presence of DPO-1, CORM-2 did not increase the amplitude or duration of currents further (Figures 8b and c), suggesting that both CO and DPO-1 acted at the same site, Kv1.5. These findings suggest that CO inhibition of Kv1.5 may be physiologically significant for atrial excitability.

## Discussion

The present study demonstrates that both native and recombinant hKv1.5 K^+^ channels are inhibited by CO. This finding adds to the growing understanding of the complexity of CO signalling in cardiac and other tissues by describing a new ion channel target for regulation.^57, 61, 62^ Significantly, it also demonstrates, for the first time, a polymodal means of channel regulation by CO that is summarized in Figure 8d. Previously, we have shown that CO-mediated augmentation of the late cardiac Na^+^ current is NO dependent and involves channel nitrosylation.^63^ Peak inward Na^+^ current inhibition by CO is also NO dependent.^64^ Inhibition of the cardiac L-type Ca^2+^ current by CO is dependent on mitochondrial ROS production but independent of NO formation.^65^ Most recently, we have shown that cardiac ERG (Kv11.1) channels are also inhibited by CO, specifically via the formation of ONOO^−^.^66^ The present study indicates that Kv1.5 is uniquely inhibited by CO acting via all of the aforementioned pathways as well as via cGMP formation, which presumably modifies channel activity via phosphorylation as suggested previously for the effects of NO.^30^

Each of these distinct means of Kv1.5 regulation by CO can be regarded as potentially important under varying physiological and pathological conditions, not only in the heart but also in the vasculature. Recent studies have provided evidence that H_2_O_2_ generated by cardiac myocytes couples cardiac metabolism to coronary flow by activating Kv1.5 channels in coronary VSMCs, which presumably leads to their relaxation (and hence vessel dilation) due to hyperpolarization and reduced Ca^2+^ influx.^33, 34, 35^ CO raises ROS levels, yet reverses augmentation of Kv1.5 by H_2_O_2_ (Figure 6). This would suggest that ROS contributing to Kv1.5 inhibition by CO are likely to be either superoxide derived from mitochondria and/or ONOO^−^ formed from superoxide and NO. It is clear that these ROS contribute to channel inhibition, whereas H_2_O_2_ has the opposite effect of augmenting current amplitudes – this suggests that Kv1.5 is differentially regulated by different oxidant species. Interestingly, CO-mediated inhibition of hKv1.5 was partly attenuated by the SOD mimetics MnTMPyP and MnTBAP (Figure 2). These agents would presumably increase H_2_O_2_ levels through superoxide dismutation, yet CO reversed the effects of exogenous H_2_O_2_ (Figure 6). This finding would suggest that CO-mediated inhibition of Kv1.5 via other mechanisms (e.g., nitrosylation or the sGC/cGMP pathway) can override augmentation by H_2_O_2_. This in turn suggests that CO levels may physiologically regulate H_2_O_2_-mediated coupling of coronary blood flow to cardiac metabolism.

Figures 2 and 3 indicate that NO formation has an important role in Kv1.5 inhibition by CO. Our findings in this regard are consistent with the study of Nunez *et al.*^30^ who demonstrated that NO inhibited recombinant hKv1.5 via nitrosylation and a cGMP-dependent mechanism. The present study shows that CO also activates these pathways by stimulating a rise in NO levels. However, NO formation does not account for all of the effects of CO as detailed here. Although molecular modelling suggested C331 and C346 as candidate cysteine residues for nitrosylation,^30^ we found that both the C331A and C346A mutants remained sensitive to inhibition by CO (Figure 5). Furthermore, both mutant channels were nitrosylated by CO, suggesting that alternative cysteines in the Kv1.5 channel are preferentially targeted for nitrosylation. Physiologically, this NO-dependent means by which CO regulates Kv1.5 activity is likely to be important both in the vasculature (particularly but not exclusively the coronary vasculature, as discussed above) and in the heart, where Kv1.5 exerts an important influence in shaping the atrial AP.^20, 21^ Kv1.5 is an important target for therapies aimed at treating AF,^23, 25^ and interestingly, maintenance or augmentation of NO bioavailability is also considered a viable approach in the treatment of AF.^41^ Our data suggest that increasing CO levels (either via stimulating HO-1 expression or via CO donors^53^) may therefore be of benefit in AF treatment via increased NO formation. However, elevated NO in the presence of superoxide can lead to the formation of ONOO^−^ and this was indeed observed in our study (Figure 4). ONOO^−^ formation is seen in AF^67, 68^ and is presumably deleterious. Increased ONOO^−^ formation contributes to shortening of the effective refractory period seen in AF, but this is unlikely to arise from inhibition of Kv1.5, as this would be expected to prolong AP duration and therefore, presumably, the effective refractory period.

A noteworthy feature of the CO-mediated inhibition of Kv1.5 was the marked slowing of its activation kinetics (see Figure 1d), which was also prominent in the mutants (Figure 5). Slowing of activation by CO appears strikingly similar to the previously reported slowing of activation caused by zinc.^69^ It would appear that this effect cannot be attributed to any particular single pathway of channel inhibition because residual inhibition caused by CO in the presence of SOD mimetics, NO or sGC inhibition or in the presence of FeTPPS to scavenge ONOO^−^ was not associated with a slowing of channel activation. This suggests that a combination of these signalling pathways is required in order to observe kinetic changes caused by CO together with channel inhibition.

To explore CO-mediated modulation of Kv1.5 in a more physiological setting, we investigated its ability to regulate K^+^ currents in the mouse atrial cell line, HL-1. Our data indicate that CO inhibits a DPO-1-sensitive K^+^ current in these cells and so presumably arises at least primarily due to activity of Kv1.5.^60^ Our data do not exclude actions of CO on other cardiac ion channels, and indeed a number of such channels are known to be CO sensitive.^61^ However, inhibition of Kv1.5 does appear to be of functional significance, as it significantly increased AP amplitude and duration in a manner that was both mimicked and occluded by DPO-1. Thus CO regulation of Kv1.5 is potentially of physiological significance for regulating atrial excitability. It may also be of pathological significance, for example, in atrial fibrillation, which is associated with increased expression of HO-1.^54, 56^

In summary, we have demonstrated that CO inhibits both native (mouse) and recombinant (human) Kv1.5 and does so via multiple signalling pathways. Tonic regulation of Kv1.5 by CO is likely to be of physiological relevance in cardiac atria as well as vascular smooth muscle, where it may regulate channel responses to other signalling factors (e.g., H_2_O_2_). The significance of Kv1.5 regulation by CO may increase under pathological conditions such as atrial fibrillation and vascular disease due to increased HO-1 expression.

## Materials and methods

### Generation and culture of HEK293 cells expressing Kv1.5

Wild-type (WT) human Kv1.5 (KCNA5) cDNA was amplified from a human foetal brain cDNA library (Clontech, Wooburn Green, Buckinghamshire, UK) using the primers: 5′-TGGAATTCACCATGGAGATCGCCCTG-3′ and 5′-GACTCGAGTCACAAATCTGTTTCCCG-3′ (Sigma-Aldrich, Gillingham, Dorset, UK) in a touchdown PCR. The ≈1.7 kb product was cloned using the CloneJET Kit (Thermo Fisher Scientific, Loughborough, Leicestershire, UK) and then subcloned into pcDNA6 (Invitrogen, Loughborough, Leicestershire, UK) using EcoRI and XhoI restriction enzymes (New England Biolabs, Hitchin, Hertfordshire, UK). At each step, clones were confirmed by Sanger sequencing (Genewiz, Bishop’s Stortford, Hertfordshire, UK).

HEK293 cells were cultured in MEM with Earle’s salts and L-glutamine, supplemented with 9% (v/v) fetal calf serum (Globepharm, Esher, Surrey, UK), 1% (v/v) non-essential amino acids, 50 *μ*g/ml gentamicin, 100 units/ml penicillin G, 100 *μ*g/ml streptomycin and 0.25 *μ*g/ml amphotericin in a humidified atmosphere of air/CO_2_ (19 : 1) at 37 °C. All cell culture reagents were purchased from Gibco-BRL (ThermoFisher Scientific) unless otherwise stated. To generate stable HEK293/Kv1.5 cell lines, cells were transfected with either a pcDNA6/Kv1.5(WT) or pcDNA6/Kv1.5(Mutant) construct using the PolyFect transfection reagent (Qiagen, Hybaid Ltd, Teddington, UK) according to the manufacturer’s instructions. Stable HEK293/Kv1.5 cell lines were achieved by antibiotic selection with blasticidin (5 *μ*g/ml, ThermoFisher Scientific), added to the medium 3 days after transfection. Selection was applied for 4 weeks (media changed every 4–5 days), after which time individual colonies were picked and seeded in T25 flasks and allowed to reach confluence. They were then transferred to T75 flasks for further culture and examination of K^+^ currents. Cells were harvested from culture flasks by trypsinization and plated onto coverslips 24–48 h before use in electrophysiological studies. Blasticidin selection was maintained throughout the entire cloning process at 5 *μ*g/ml and then subsequently reduced to 2.5 *μ*g/ml in all subsequent passages of cells once stable clones had been positively identified.

The C331A and C346A mutations were introduced into WT human Kv1.5 (hKv1.5, in pcDNA6) using the Quik-Change Site-Directed Mutagenesis Kit (Stratagene, Cheadle, UK) according to the manufacturer’s instructions. All constructs were verified by DNA sequence analysis before transfection.

### Culture of HL-1 cells

HL-1 atrial cardiomyocytes were maintained in Claycomb media (Sigma, UK) supplemented with batch-specific 10% FBS (Sigma, Gillingham), 1% penicillin/streptomycin (Invitrogen), 0.1 mM norepinephrine (Sigma) and 2 mM l-glutamine (Invitrogen). Cells were cultured in flasks, or on coverslips, pretreated with 0.02% Bacto gelatin (Fisher Scientific, Loughborough, UK) and 0.5% fibronectin (Invitrogen).

### Exposure to CO

CO was applied to HEK293 cells and HL-1 cells via the CORM, CORM-2. CORM-2 was prepared no longer than 1 h before use by dissolving in dimthylsulphoxide (DMSO) at a stock concentration of 30 mM so that dilution into perfusate or other solutions in which cells were maintained (e.g., electrophysiology, imaging, biotin switch assay) usually resulted in DMSO levels of no more than 1 : 1000. iCORM, which served as a negative control, was prepared by dissolving CORM-2 identically and leaving in perfusate solution for 2 weeks prior to use, by which time all CO was released and lost from solution.

### Electrophysiology

Coverslips with cultured cells were transferred from the incubator into a recording chamber mounted on the stage of an Olympus CK40 inverted microscope (Olympus, London, UK) and continually perfused with bath solution (2–4 ml.min^–1^) containing the following: 140 mM NaCl, 4 mM KCl, 2 mM CaCl_2_, 1 mM MgCl_2_, 5 mM glucose, buffered with 10 mM HEPES, pH 7.4. Single cells were selected for whole-cell patch-clamp experiments at 22±1 °C. Pipettes were filled with intracellular solution (140 mM KCl, 10 mM NaCl, 4 mM MgCl_2_, 20 mM EGTA, 10 mM HEPES, pH 7.2) and had a resistance of 4–6 MΩ. Whole-cell voltage-clamp or current-clamp experiments were recorded, digitized and stored with an Axopatch 200B amplifier, Digidata 1322A and pCLAMP 10 respectively (Molecular Devices, Union City, CA, USA).

Series resistance was compensated by 70–90%. If a significant increase in series resistance occurred (>20%), the experiment was terminated. Leak currents were subtracted using the P/4 protocol in the pCLAMP software and voltage-clamp signals were sampled at 50 kHz and low-pass filtered at 20 kHz; *I*–*V* relationships were measured by stepping from a holding potential of −90 mV to voltages between −60 and +80 mV in 10 mV increments for 500 ms. Time-series experiments were measured using a single pulse protocol stepping from −90 to +50 mV for 100 ms every 5 s.

HL-1 spontaneous APs were acquired in gap-free mode with no current injected. IK_ur_ was recorded as described previously^70^ using a 100 ms prepulse to +40 mV to inactivate *I*_to1_, followed by a 150 ms test pulse from −50 to between −40 and +50, then to −30 mV.

Offline analysis was performed using the data analysis package Clampfit 10.0 (Molecular Devices, Foster City, CA, USA), and subsequent fitting and statistical analysis was undertaken using GraphPad Prism 7 (GraphPad Software, La Jolla, CA, USA). Results are presented as means±S.E.M., with ‘*n*’ representing the number of experiments performed. Statistical significance was evaluated using unpaired Student’s *t*‐tests where differences were considered significant when the *P*-value was <0.05.

### Biotin-switch assay

Detection of S-nitrosylated hKv1.5 was performed using the biotin-switch assay followed by western blotting as previously described.^71^ Briefly, HEK293 cells expressing WT or mutant (C331A or C346A) Kv1.5 were harvested and lysed in a non-denaturing solution (in mM: 50 Tris-HCl, 300 NaCl, 5 EDTA, and 1% Triton-X). Extracts were adjusted to 0.5 mg/ml and incubated with CORM-2 (30 *μ*M) for 15 min at 37 °C; inactive CORM (iCORM, 30 *μ*M) and DMSO were used as controls.

CORM-2 and iCORM were removed and buffered exchanged using desalting spin columns (Thermo Fisher Scientific). From this point, all procedures were carried out in the dark. Lysates were incubated in blocking buffer (in mM: 225 HEPES, 0.9 EDTA, 20 methyl methanethiosulfonate (MMTS), and 2.5% SDS, pH 7.4) for 20 min at 50 °C with agitation. Lysates were subjected to buffer exchange to remove MMTS and eluted in HENS buffer (mM: 250 HEPES, 1 EDTA, and 1% SDS, pH 7.4) and incubated with 1/3 volume of *N*-[6-(biotinamido)hexyl]-3′-(2′-pyridyldithio) propionamide (biotin-HPDP, Pierce, Loughborough, UK) and ascorbate (1 mM) for 1 h at room temperature, followed by buffer exchange to remove biotin–HPDP from the samples. Unless otherwise stated, all buffers were supplemented with protease inhibitor cocktail tablets (Roche, Welwyn Garden City, UK).

Biotinylated proteins were detected via western blotting as described previously.^28^ Protein samples were prepared without reducing agents and were not boiled before electrophoresis to prevent the reversal of cysteine biotinylation and non-specific biotin–HPDP reactions, respectively.

### Fluorescence detection of nitric oxide (NO) and peroxynitrite (ONOO^−^)

Cells were plated on coverslips and allowed to grow for 48 h at 37 °C in a humidified atmosphere containing 95% air and 5% CO_2_ before being preincubated for 1 h with DAF-2 diacetate (5 *μ*M; Invitrogen), prepared in the following extracellular solution: 140 mM NaCl, 4 mM KCl, 1.5 mM CaCl_2_, 2 mM MgCl_2_, 10 mM HEPES, 10 mM glucose, pH 7.4). Cells were then gently washed twice with extracellular solution and then left for at least 15 min in an incubator to allow the hydrolysis of DAF-2 diacetate into the free NO-sensitive free acid form (DAF-2).

Fragments of coverslip with attached cells were placed on a Zeiss (Oberkochen, Germany) laser scanning confocal microscope (LSM 510) fitted with a × 40 oil immersion lens (Zeiss Plan Neofluar, refractive index of 1.3) and continuously perfused with extracellular solution at a rate of 0.5 ml/min. DAF-2 loaded cells were excited with the 488-nm line of a 20-mW diode laser (attenuated by ∼90%), and emitted fluorescence was measured at >515 nm. *x*–*y* images were obtained from isolated cells at 1 min intervals to minimize photobleaching using the Zeiss AIM software. Experimental settings were identical in all test conditions and each experiment was repeated five times. Fluorescence intensity of isolated cells was analysed using the ImageJ software (Laboratory for Optical and Computational Instrumentation, Madison, WI, USA) and data are presented as means±S.E.M.

To detect ONOO^−^, cells grown on coverslips were incubated for 1 h at 37 °C with APF (10 *μ*M) dissolved in HEPES-buffered saline. For L-NAME or FeTPPS experiments, cells were preincubated with either drug at the same time as the APF treatment. Coverslips fragments with cells attached were placed in a chamber (as a static bath set-up) filled with 200 *μ*l of HEPES-buffered saline containing 10 *μ*M APF. Changes in fluorescence intensity were measured over 10 min using a ZEISS (Oberkochen) laser-scanning confocal microscope (LSM 510). APF was excited at 488 nm and emission monitored at 510 nm, and images were obtained using the Zeiss AIM software. All settings were identical for control and test conditions.
